# ORAL CONCURRENT SESSION 4: Basic and Translational Science

**DOI:** 10.1002/pmf2.12011

**Published:** 2025-01-05

Abstracts 47 – 56

FRIDAY

January 31, 2025

1:30 PM – 4:00 PM

Aurora Ballroom A

MODERATORS

Michael House, MD

Jamie O. Lo, MD

